# Sex differences in the genetic architecture of dilated cardiomyopathy

**DOI:** 10.1016/j.jacc.2025.04.068

**Published:** 2025-08-05

**Authors:** Massimo Mangino, Kathryn A McGurk, Pantazis Theotokis, Rachel J Buchan, Clare R Stockwell, Prisca K Thami, Emma Jennings, Elaine Wan, Catherine Enright, Ahmed Adlan, Tamim Akbari, John Baksi, Paul JR Barton, Colin Berry, Gerry Carr-White, Amedeo Chiribiri, Rob Cooper, Abhishek Dattani, Andrew S Flett, Roy S Gardner, John P Greenwood, Brian P Halliday, Daniel J Hammersley, David C Hutchings, Masliza Mahmod, Benjamin Marrow, Gerry P McCann, Manish Motwani, Stefan Neubauer, Declan P O’ Regan, Stephen P Page, Antonis Pantazis, Charles Peebles, Betty Raman, Peter P Swoboda, Amanda Varnava, Luigi Venetucci, Hugh Watkins, David Wright, GO-DCM Investigators, Sanjay K Prasad, Stuart A Cook, James S Ware, Upasana (Paz) Tayal

**Affiliations:** 1 National Heart Lung Institute, Imperial College London, London, UK; 2 Kings’ College London, London, UK; 3 MRC Laboratory of Medical Sciences, Imperial College London, London, UK; 4 Royal Brompton & Harefield Hospitals, Guy’s and St Thomas’ NHS Trust, London, UK; 5 University of Manchester, Manchester, UK; 6 Manchester Heart Institute, Manchester University NHS Foundation Trust, Manchester, UK; 7 School of Cardiovascular and Metabolic Health, University of Glasgow and NHS Golden Jubilee National Hospital, Glasgow, UK; 8 Guy’s and St Thomas’ NHS Foundation Trust, London, UK; 9 University of Liverpool, Liverpool, UK; 10 Liverpool Heart and Chest Hospital, Liverpool, UK; 11 Liverpool John Moores University, Liverpool, UK; 12 Department of Cardiovascular Sciences, University of Leicester, and the NIHR Biomedical Research Centre, Glenfield Hospital, Leicester, UK; 13 University Hospital Southampton NHS Trust, Southampton, UK; 14 Leeds Teaching Hospitals NHS Trust, Leeds, UK; 15 Baker Heart and Diabetes Institute, Melbourne, Australia; 16 University of Oxford, Oxford, UK; 17 Institute of Clinical Sciences, Imperial College London, London, UK; 18 The University of Leeds, Leeds, UK; 19 Imperial College Healthcare NHS Trust, London, UK; 20 The George Institute for Global Health, London, UK

**Keywords:** Sex differences, DCM, desmoplakin

## Introduction

Dilated cardiomyopathy (DCM) has a monogenic aetiology in up to a third of cases^1^. It appears to affect twice as many males compared to females. This sex difference is observed in both monogenic autosomal cases and DCM without an identified monogenic cause. This could be due to ascertainment bias, e.g. differences in diagnostic criteria, or sex differences in disease susceptibility, with males at risk or females protected. It is not known whether specific genetic subtypes manifest sex-differences in penetrance. We have extensively characterised the genetic architecture of DCM^2, 3^. In this study we investigated whether there are sex differences in the underlying genetic architecture of DCM, indicating gene-specific influences of sex on disease susceptibility.

## Methods

### DCM cohort

Adult patients with DCM (n=902) were prospectively recruited from 11 centres in the United Kingdom. DCM was diagnosed using reference criteria of left ventricular or biventricular systolic dysfunction and dilatation not explained solely by loading conditions or coronary artery disease^4^. The study was approved by the NHS Health Research Authority (REC reference: 18/LO/0692).

Whole genome sequencing (WGS) was performed with a 30x coverage. Variant calling was performed using Haplotype Caller implemented in Genome Analysis Toolkit (v3.4^5^). Variants with genotyping call rates <90%, QualByDepth <6, Hardy-Weinberg equilibrium *P*-values <1.0×10^-6^ and samples with call rates <90%, excess heterozygosity, chromosomal sex ambiguity, and relatedness (up to second-degree) were excluded. Variant annotation was performed with Ensembl Variant Effect Predictor (VEP; v111), with variant consequence defined against Matched Annotation from NCBI and EMBL-EBI (MANE) transcripts : i) predicted loss of function (pLoF; nonsense, frameshift or essential splice site variants), ii) missense (missense or in-frame insertion/deletion variants), and iii) protein-altering variants (PAVs; pLoF and missense combined). For titin (*TTN*), only pLoF variants altering constitutively expressed cardiac exons were included. Burden analyses were performed with Fisher’s exact tests on twelve genes defined as “definitive” DCM-related genes in CardiacG2P^6^. For each gene, the combined frequency of rare variants (minor allele frequency (MAF) <0.01% within each gnomAD population^7^), restricted to variant classes consistent with the mechanism of disease for that gene, were compared between males and females. Genes with insufficient sample size (defined as fewer than five samples carrying the rare variant in the combined male and female dataset) were excluded by the analysis. Odds ratios (ORs) are reported with 95% confidence intervals. To correct for multiple testing, P-values were adjusted using the Benjamini and Hochberg method, with P_FDR_ <0.01 considered statistically significant.

### P*opulation evaluation of sex-stratified DCM risk associated with* DSP *variants*

To validate DSP results, sex-stratified association tests were performed in UK Biobank (UKB). Protein altering variants within the region of the desmoplakin gene (*DSP)* were analysed from the whole exome sequencing (WES) data of 469,397 UKB participants. Variant annotation and classification was performed consistent with methods for the DCM cohort. Most truncating variants (pLoF) in DSP were pathogenic/likely pathogenic (P/LP) and more likely to be causative for disease than non-truncating variants, so only pLoF variants were analysed. Variants in the MANE transcript were identified with a MAF <0.01% (popmax) in gnomAD (v3.12) and UKB, and passed UKB quality control. A sex-stratified association with ICD-10 coded DCM was tested using Fisher’s exact test.

### *Sex-stratified estimates of DCM penetrance of* DSP *variants*

Population penetrance was estimated using our previously reported cross-sectional approach that compares aggregated allele frequencies in diagnostic cases^8^ against population frequencies (167,478 UKB carriers; 75,727 males, 91,751 females) for *DSP* and other DCM-related genes.

## Results

From 902 patients with a diagnosis of DCM recruited, 830 unrelated individuals (756 European, 25 African/African American, 9 Admixed American and 40 South Asian) had eligible WGS data meeting quality control criteria. Of these, 274 were female (33%). The cohort median age was 56.0 years (IQR 45.0-65.0), and the median left ventricular ejection fraction was 37% (IQR 26-47).

When comparing genetic architecture between males and females, *DSP* was the only gene that accounted for a different proportion of cases in males versus females, with *DSP* variants more prevalent in female patients (Table 1; *DSP* PAV 7.3% in females, 2.5% in males, OR 3.04: 95% CI: 1.44-6.63; *DSP* pLOF 2.2% in females, 0.36% in males, OR 6.19: 95% CI 1.1-63). In contrast, there was no significant difference between males and females in the frequency of variants in disease-associated variant classes for *LMNA, RBM20, SCN5A, TTN, FLNC, DES*, or *MYH7* (Table 1). Four genes (*BAG3, TNNC1, TNNT2* and *PLN*) were excluded from the analysis because the sample size was insufficient to perform the sex stratified analysis. The findings were consistent in sensitivity analyses restricted to European ancestry participants (N=756), with PAVs in *DSP* still enriched in female patients compared with males (OR 3.40: 95%CI 1.56-7.64).

Amongst 469,397 participants in UKB, 1,304 had a diagnosis of DCM (0.28%); 387 of whom were female (30%). UKB results validated the findings observed in the main cohort. *DSP* pLoF variants were enriched in UKB females with DCM (*DSP* PAV 5.2% in females, 2.9% in males, OR 1.8, CI 0.94-3.4; *DSP* pLOF 2.3% in females, 0.22% in males, OR 10.9: 95% CI: 2.2-104.0).

Estimating sex-stratified penetrance, the overall population penetrance^8^ of *DSP* pLoF was estimated as 0.08 (95%CI 0.05-0.12) for DCM, with sex-stratified estimated population penetrance in females of 0.11 (95%CI 0.06-0.20), and male penetrance of 0.02 (95% CI 0.01-0.07). Additionally, TTNtvs had significantly higher penetrance in males (Table 1).

## Discussion

This study shows that pLoF variants in desmoplakin account for a larger proportion of female patients with DCM compared with male patients and variants in *DSP* confer a greater risk of DCM in females.

These results have clinical significance. *DSP* variants are associated with adverse outcomes compared to non-*DSP* variants and female sex is an adverse prognostic marker in *DSP* cardiomyopathy, potentially identifying a higher risk sub-group of female patients with DCM^9^.

Most DCM genes are not found on the sex chromosomes, so the prevalence of variants does not differ between the sexes. We show that variants in *DSP* show higher penetrance in females and truncating variants in *TTN* show higher penetrance in males. Variants in other DCM genes did not appear to account for a different proportion of cases amongst males and females. The DCM male preponderance may be due to sex-specific factors that modulate the risk of non-desmoplakin DCM variants and modulate the risk of non-monogenic DCM.

Potential limitations include the limited ancestral diversity, so we were unable to evaluate sex and ancestry stratified differences. Further research is required to understand the genetic, environmental and reproductive factors^10^ that underpin the unique increased female susceptibility to desmoplakin variants.

## Figures and Tables

**Table 1:** Comparison of variant frequencies in male and female DCM patients, and population penetrance estimates. (i) Desmoplakin protein-altering variants were significantly enriched in female patients compared to males. OR, odds ratio of the comparison of male and female carrier counts; FDR, false discovery rate. Burden analyses were performed with Fisher’s exact tests. Association tests with P_FDR_ <0.01 were considered statistically significant. The variant classes were restricted to *TTN, FLNC* predicted loss of function (pLoF); *DES, MYH7* missense (ms); *DSP, LMNA, RBM20, SCN5A* protein-altering (PAV; both missense and pLoF). (ii) Penetrance, the probability of DCM in variant carriers by gene as estimated via cross-sectional approach, using previously described methods and data^8^, was estimated for pLoF variants. Estimates are only given for *DSP* and *TTN* as the genes with sufficient pLoF variants; missense variants are excluded to avoid conflating penetrance with the proportion of benign versus pathogenic variants. *DSP* pLoF are more penetrant in females, *TTN* pLoF variants are more penetrant in males.

*(i) Burden analysis in DCM cohort*						
	Females (N=274)		Males (N=556)		OR (95%CI)	P value (FDR-adjusted)
Gene	N	%	N	%		
***FLNC***	2	0.73	4	0.72	1.01 [0.09 - 7.13]	1.00
***TTN***	37	13.50	105	18.88	0.67 [0.43 - 1.02]	0.12
***DES***	2	0.73	6	1.08	0.67 [0.07 - 3.80]	1.00
***MYH7***	10	3.65	12	2.16	1.72 [0.65 - 4.40]	0.75
***DSP***	20	7.30	14	2.52	3.04 [1.44 - 6.63]	0.0092
***LMNA***	7	2.55	8	1.44	1.79 [0.55 - 5.73]	0.54
***RBM20***	5	1.82	7	1.26	1.46 [0.36 - 5.39]	0.54
***SCN5A***	6	2.19	8	1.44	1.53 [0.43 - 5.10]	0.54
***(ii) Population penetrance estimates***						
**Gene**		**Female penetrance** **[95% CI]**			**Male penetrance** **[95% CI]**	
***TTN* pLoF**		0.06 [0.04 - 0.09]			0.18 [0.14 - 0.25]	
***DSP* pLoF**		0.11 [0.06-0.20]			0.02 [0.01-0.07]	

## Data Availability

Requests for the extracted data will be considered upon request to the corresponding author. Supplementary table of variants used in this analysis is available at: DOI 10.5281/zenodo.15270524 (created 23.4.25; accessed 23.4.25) ^11^

## Clinical Question

Dilated cardiomyopathy appears to be more common in males, even in genetic forms, but it is not clear if this is due to under-diagnosis in females, or whether males are truly at higher risk. Specifically, it is not known whether specific genetic subtypes manifest sex-differences in penetrance.

### Main finding

- Deleterious variants in the desmoplakin gene account for a larger proportion of female patients with dilated cardiomyopathy compared with male patients.
- Variants in desmoplakin confer a greater risk of dilated cardiomyopathy in females.
- These results have clinical significance. Desmoplakin variants are associated with adverse outcomes compared to non-desmoplakin variants and female sex is an adverse prognostic marker in desmoplakin cardiomyopathy, potentially identifying a higher risk sub-group of female patients with dilated cardiomyopathy.
